# Screening University Students for Health Checks With an Electronic Health Questionnaire in Finland: Protocol for a Retrospective, Register-Based Cohort Study

**DOI:** 10.2196/14535

**Published:** 2020-01-29

**Authors:** Susanna Paldanius, Noora Seilo, Kristina Kunttu, Reija Autio, Minna Kaila

**Affiliations:** 1 Faculty of Medicine and Health Technology Tampere University Tampere Finland; 2 Science Centre Pirkanmaa Hospital District Tampere Finland; 3 Tampere City Student Health Care Tampere Finland; 4 Finnish Student Health Service Helsinki Finland; 5 Faculty of Social Sciences Tampere University Tampere Finland; 6 Public Health Medicine University of Helsinki Helsinki Finland; 7 Helsinki University Hospital Helsinki Finland

**Keywords:** electronic health questionnaire, health check, screening, students, student health services, digitalization, register study, preventive health services

## Abstract

**Background:**

Health questionnaires and health checks are an established part of preventive health care services in Finland. However, only very limited research of these has been conducted. The Finnish Student Health Service (FSHS) provides primary health care services to all bachelor’s and master’s degree university students (approximately 134,500 students) in Finland. FSHS’s statutory health examination process of university entrants includes an electronic health questionnaire (eHQ) and, based on the students’ eHQ responses, a subsequent health check if necessary. To our knowledge, no previous studies have been published on the use of questionnaires for screening students for general health checks.

**Objective:**

The general aim of the study is to evaluate the health examination process of university entrants. The objectives are to determine how students’ self-reported health in the eHQ and participation in the health examination process are associated with graduation, mental health problems, and the use of student health care services.

**Methods:**

This is an ongoing, nationwide, retrospective, register-based cohort study with a 6-year follow-up. The study population is the cohort of university entrants (N=15,723) from the 2011-2012 academic year. These students were sent the eHQ, which consisted of 26 questions about health, health habits, social relations, and studying. Based on the eHQ responses, students were referred to one of the following interventions: (1) a health check, (2) an appointment other than a health check (eg, physiotherapy), or (3) electronic feedback to support a healthy lifestyle, when the other interventions were not necessary. Multiple comparisons will be made within these groups using logistic regression. The primary outcome variables are graduation, having a mental health problem, and attending a health check. The use of FSHS health care services will be studied with the cluster analysis method. The data have been obtained from three nationwide registers: the eHQ register, the medical records of FSHS, and the Higher education achievement register. The data have been linked using personal identity codes.

**Results:**

As of August 2019, the data collection and processing are complete and the statistical analyses are in progress. Preliminary results are expected in autumn 2019. Further publications are expected in 2020, and two PhD theses are expected to be completed by the end of 2022.

**Conclusions:**

Studying practical procedures in primary health care is highly important for resource allocation and the development of evidence-based processes. This study will be the first to assess the usage of a health questionnaire in screening students for health checks. The findings of this study will contribute to the field of preventive health care. The main practical implication is the development of the FSHS’s health examination process. We hypothesize that participation in the health examination process enhances academic achievement and the detection of university students’ mental health problems early on in their studies.

**International Registered Report Identifier (IRRID):**

DERR1-10.2196/14535

## Introduction

The well-being of university students has raised concerns [1,2]; the awareness of students’ mental health problems, especially, has grown [3-5]. In Finland, health questionnaires and health checks are essential preventive measures for the early detection of health concerns in student health care [6,7]. However, only very limited research has been conducted on these measures [8-10]. In general, the evidence on the effects of health checks is inconclusive, both in the student and the general populations [8,11-15].

Finland has a strong tradition of offering preventive health care services to its residents (see Multimedia Appendix 1) [16]. Student health care is a part of this statutory preventive health care provision (see Textbox 1) [7]. The promotion of the health and study ability of the students at the level of individuals and communities forms a central part of student health care services (see Multimedia Appendix 2) [17].

The Finnish Student Health Service (FSHS) provides student health care services to all bachelor’s and master’s degree university students in Finland (approximately 134,500 students) [18,19]. FSHS has provided health checks to all university entrants since the 1970s. However, in the beginning of the 21st century, universal health checks were identified to be an area for development due to low participation rates. As a solution, FSHS developed a two-stage health examination process. The process included an electronic health questionnaire (eHQ) and, based on the students’ eHQ responses, a subsequent health check if necessary. The eHQ includes 26 questions about health, health habits, social relations, and studying (see Multimedia Appendix 3). Providing a digitalized health questionnaire to all university entrants instead of universal health checks was believed to increase the number of students reached. Further, the process was aimed to facilitate identification of students with health problems and to target health checks to these students.

The two-stage health examination process was developed by following the ideology of the plan-do-study-act cycle [20]. Feasibility of the eHQ was tested in 2005 [9]. A pilot study of the process was conducted in 2008 [21]. Based on the results of these studies, the eHQ was further developed. The health examination process was implemented nationally in 2009. The data collected over time now enable studying the process again.

To our knowledge, no previous studies have been published on the use of a questionnaire for screening students for general health checks. However, multiple studies exist about different questionnaires that are used to detect specific symptoms [22-24] or to evaluate health behavior and social conditions [25-28] in student populations. The eHQ aims to provide an overview of the health and well-being of university entrants rather than to identify specific conditions.

The general aim of this study is to evaluate the health examination process of FSHS. The specific research questions are as follows:

1. Is responding to the eHQ and attendance at the health check associated with completing a bachelor’s or master’s degree in the 6-year follow-up?
2. How are mental health problems associated with completing a bachelor’s or master’s degree in the 6-year follow-up?
3. How are responding to the eHQ and attendance at the health check associated with the use of FSHS’ health care services?
4. How are university entrants’ responses to the eHQ questions associated with:
  - completing a bachelor’s or master’s degree?
  - health check attendance?
  - mental health problems?
  - the use of FSHS health care services?

**Student health care services of university students according to the Finnish Health Care Act [7].**

- Triennial checks on health and safety in educational institutions and welfare promotion among learning communities
- The monitoring of students’ health, welfare, and fitness to study, including a health questionnaire during the first year of study leading to a health check if necessary
- The provision of health and medical care services for students, including mental health and substance abuse services, advice on sexual health, and oral health care
- Early identification of any special needs and tests required by students, support, and, if necessary, referral to further tests or treatment

To the best of our knowledge, this type of health examination process will be studied for the first time. We hypothesize that the health examination process enhances university students’ academic achievement and the early detection of mental health problems.

## Methods

### Design

**Figure 1:**
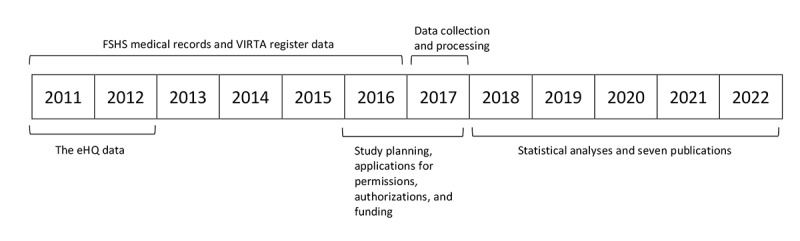
The study timeline. eHQ: electronic health questionnaire; FSHS: Finnish Student Health Service; VIRTA: higher education achievement register.

This is a nationwide, retrospective, register-based cohort study with a 6-year follow-up (see Figure 1). The study population is the cohort of Finnish university entrants from the 2011-2012 academic year (N=15,723). Data from three nationwide registers have been linked and will be analyzed in order to answer the research questions.

### Study Population

As stated, the study population is comprised of university entrants who enrolled in the 2011-2012 academic year (N=15,723). According to Statistics Finland, 2.7% (N=145,800) of the Finnish population were studying for a bachelor’s or master’s degree in one of the 13 universities in 2012 (see Table 1); 19% of men and 26% of women in the 19-21-year-old age class had entered a university [29]. In 2017, the median time to complete a bachelor's degree was 3.8 years and the median time to complete a master’s degree, including a bachelor’s degree, was 5.9 years [30].

The Finnish Student Health Survey has been conducted every 4 years since 2000 [31]. The survey indicates that Finnish university students are, in general, healthy. In 2016, 75% reported good or very good overall well-being [31]. However, mental health problems are a significant challenge in the Finnish student population. Every third student reported mental health problems in a 12-item general health questionnaire, and 7% were identified to be burnt out according to the study burn-out inventory [24,31].

**Table 1:** University students in Finland in 2012 according to Statistics Finland^a^.

Students’ degree status	Total, N	Female, n (%)	Male, n (%)
Studied either a bachelor’s or master’s degree	144,279	76,979 (53.35)	67,300 (46.65)
Started a bachelor’s degree	15,218	8624 (56.67)	6594 (43.33)
Started a master’s degree	4874	2538 (52.07)	2336 (47.93)

^a^Statistics Finland provides publicly available statistical information.

### The Health Examination Process of the Finnish Student Health Service

**Figure 2:**
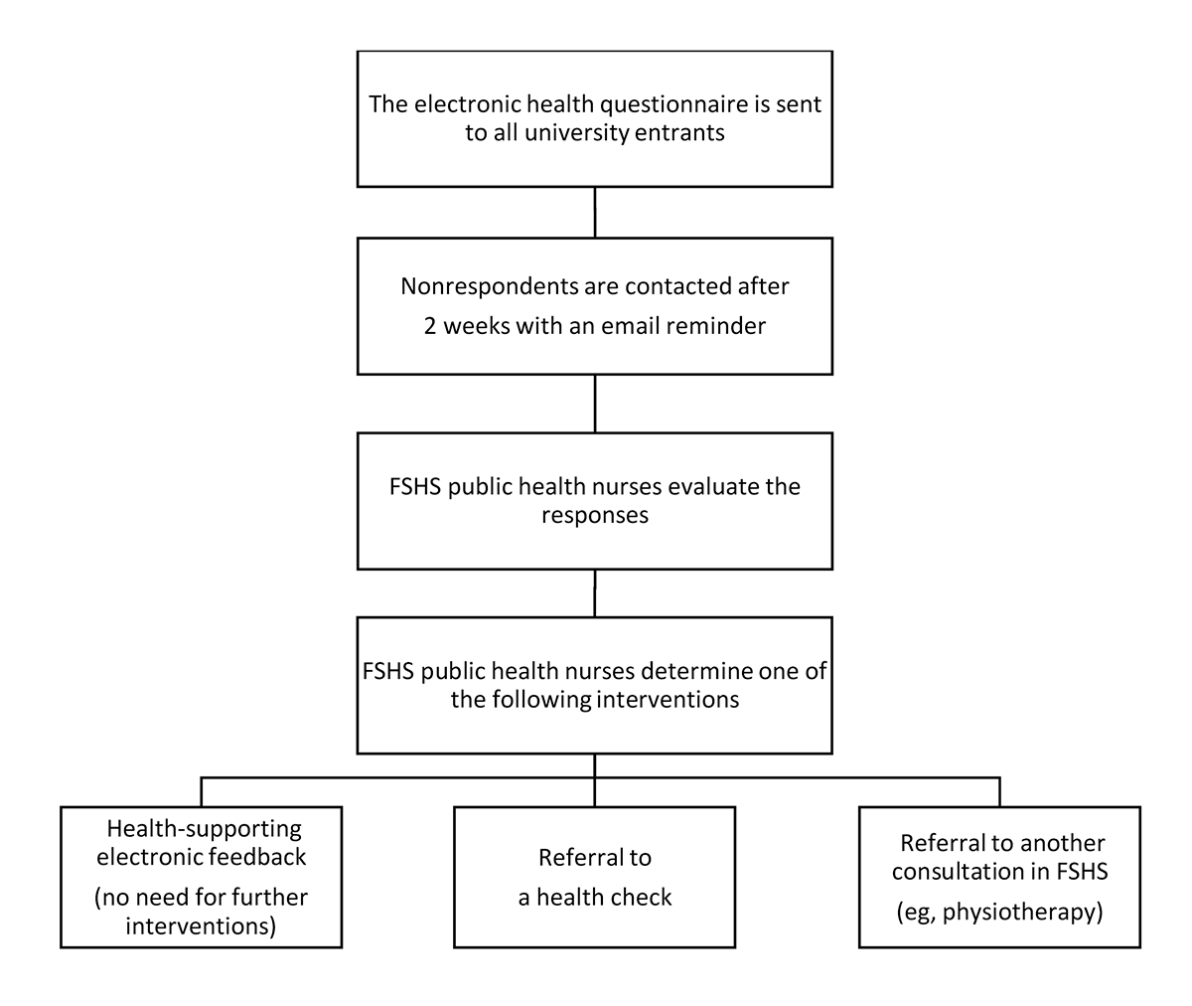
The health examination process of the Finnish Student Health Service (FSHS) in the 2011-2012 academic year.

The health examination process of FSHS includes an eHQ sent to all university entrants and a subsequent health check if necessary (see Figure 2). The process produces data about students’ health and risk behaviors for FSHS. The data are used to develop student health care services and study environment.

The purpose of the eHQ is to give an overview of students’ well-being and to identify students who have potential risk factors for study ability (see Multimedia Appendix 2). Students with potential risk factors are offered a chance for a health check conducted by a public health nurse. In the health check, the eHQ serves as a basis for discussion. In addition to being a screening tool, the eHQ is thought to be an intervention itself by motivating students to consider their health behavior.

It has been suggested that to detect health problems associated with academic functioning, a health questionnaire in student health care should include questions about social support; general, physical, and psychological health; study-related issues; help-seeking behavior; and life events in the past [32]. The eHQ covers all these subjects except for past life events.

The eHQ consists of 26 questions about physical and mental health, social relations, and studying (see Multimedia Appendix 3). It includes questions about self-rated health, long-term diseases, and recurrent symptoms. Health habits, such as exercising, eating habits, sleeping, and substance abuse, including the Alcohol Use Disorders Identification Test (AUDIT), are reported [33]. Mental health-related questions cover, for example, questions about usual state of mind and loneliness. Most of the questions are adapted from validated questionnaires or from Finnish population surveys. However, the eHQ as a whole has not been validated.

The invitations to answer the eHQ were sent in clusters via email during the 2011-2012 academic year. To fill the eHQ, the students signed into a separate program protected by strong electronic identification [34]. The students who were referred to a health check were responsible for making the appointment themselves. Responding to the eHQ and attending the health check were voluntary for students. In the study, we will compare eHQ respondents with nonrespondents, and attendees to the health check with nonattendees, in terms of the research questions.

**Figure 3:**
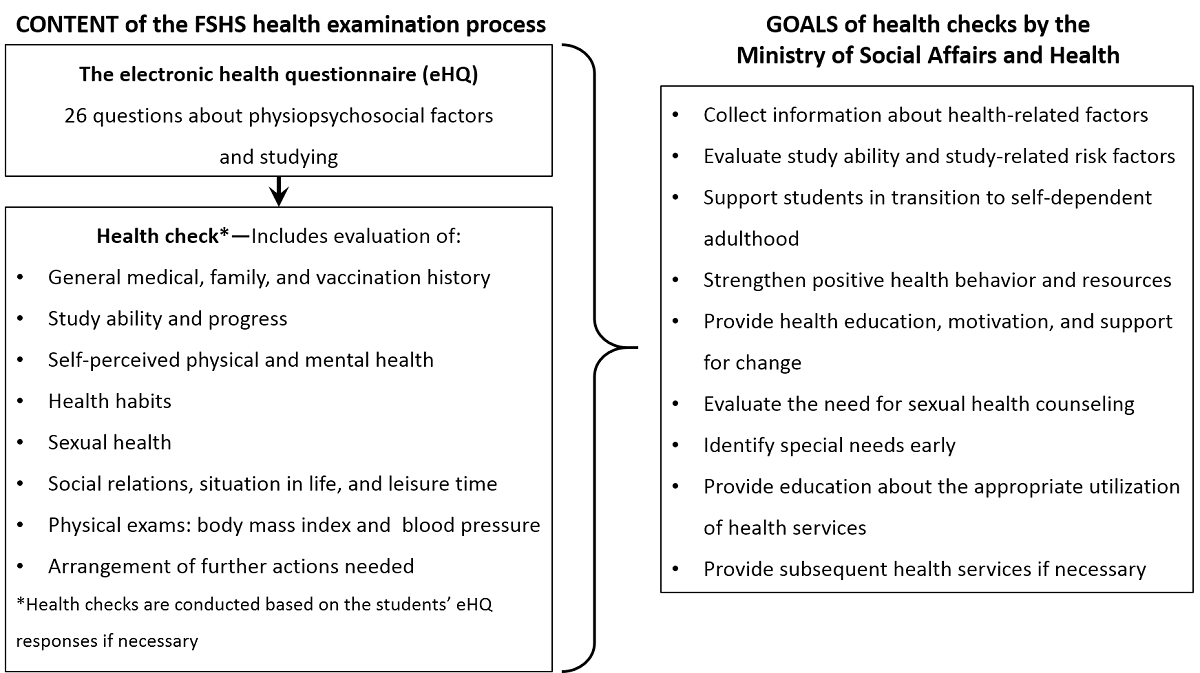
The content of the health examination process defined by the Finnish Student Health Service (FSHS) and the goals of health checks in student health care according to the Ministry of Social Affairs and Health.

The general goals of the health checks are defined by the Ministry of Social Affairs and Health, whereas the content of the checks is undefined (see Figure 3) [6,17]. Therefore, FSHS has defined the content of the health checks for university students (see Figure 3).

### Data Sources

The data have been obtained from three nationwide registers: the eHQ register, the medical records of FSHS, and the *Higher education achievement register* (VIRTA) owned by the Ministry of Education and Culture [35].

The response data to the annual eHQ accumulate within the eHQ register, which is a separate part of the medical records of FSHS. The register is owned and managed by FSHS. The response data have been obtained for the 2011-2012 academic year.

The FSHS’s medical records include systematic documentation on the students’ medical history and care at FSHS. The study data include the following: (1) primary reasons for the encounters, (2) primary diagnoses, (3) number of encounters, and (4) profession of the health care professional involved in the encounter for the 2011-2017 period. The outcome variables *mental health problem* and *health check attendance* are derived from the medical records data. Mental health problems are identified based on the 10th revision of the International Statistical Classification of Diseases and Related Health Problems (ICD-10) and the International Classification of Primary Care, Second edition (ICPC-2) classifications [36,37].

VIRTA is the Higher education achievement register of the national data warehouse for higher education. The register includes, for example, records of graduations of all Finnish higher education institutions. The information about students’ graduations has been obtained for the 2011-2017 period. The outcome variable *graduation* was obtained from the VIRTA data.

The data have been linked using Finnish personal identity codes. All Finnish citizens and permanent residents have personal identity codes administered by the Population Register Centre [38]. The code is individual to its holder and remains unchanged throughout the holder’s lifetime [38].

### Statistical Analyses

To describe the data, the frequencies, percentages, and medians with interquartile ranges will be calculated. For the preliminary analysis, chi-square tests will be employed to detect the associations between the categorical variables. Further, the normally distributed data will be analyzed with *t* tests and analyses of variance. In the cases where data are not normally distributed, the Mann-Whitney U test and the Kruskall-Wallis test for detecting the differences between the groups will be utilized. Multiple comparisons will be performed with the Bonferroni method.

In research questions 1, 2, and 4 a-c, multiple logistic regression will be the main analysis method to account for the associations between explanatory and outcome variables. The outcome variables in the regression analyses will be *graduation*, *having a mental health problem*, and *attending the referred health check*. The main explanatory variables will be students’ responses to the eHQ. In addition, demographic factors (ie, age, sex, and field of study) will be accounted for.

In research questions 3 and 4 d, the use of FSHS health care services will be analyzed with clustering analysis to detect the patterns of how the students are using the services. The patterns will then be analyzed with the explanatory variables in order to find the associations between service use and other variables.

Comparisons in the study will be made between eHQ respondents and nonrespondents, health check attendees and nonattendees, graduates and nongraduates, and students who have and do not have mental health problems.

The analyses will be carried out using IBM SPSS Statistics for iOS and Windows, version 25.0 or later (IBM Corp), and R, version 3.6.1 (The R Foundation), with suitable packages [39].

## Results

### Schedule

As of August 2019, the data collection and processing are complete and statistical analyses are in progress. Preliminary results are expected in autumn 2019. Further publications are expected in 2020, and two PhD theses are expected to be completed by the end of 2022 (see Figure 1).

### Ethics and Governance

The study is being conducted under the guidelines of the Finnish National Board on Research Integrity [40]. The study has been ethically reviewed by the Ethics Committee of the Tampere Region (reviews 2/2017 and 23/2017). The review was affirmative.

The study has been evaluated and authorized by the Finnish National Institute of Health and Welfare, which authorizes the research use of confidential data in Finland (Dnro THL/1364/5.05.00/2017) [41]. The study has received permission from the FSHS to conduct research. All 13 Finnish universities have given permission for their part to use the Higher education achievement register. A risk assessment and data protection plan has been delivered to the Finnish office of the data protection ombudsman.

## Discussion

This study is the first to assess the usage of a health questionnaire in screening students for health checks. In addition, the study explores the eHQ in identifying the students who have mental health problems and the effects of attending the health check.

The strengths of the study are its high-quality nationwide register data with good coverage and the high percentage of completed questionnaires from the respondents. The register data enable the assessment of the whole cohort of university entrants with a relatively long follow-up. Conducting register-based studies in Finland is feasible due to the unique identity codes that enable data linkage between the registers and individual-level analyses [42].

The limitations of register-based studies, in general, should be considered. Even though it has been found that Finnish administrative registers are of high quality, missing or incorrectly recorded data are always a possibility [42]. In this study, the medical records might include missing or false data due to the possibility of human error. In addition, it might also be counted as a limitation that the eHQ is not a validated questionnaire.

It is valuable to study primary health care practice with respect to resource allocation and conducting evidence-based processes. The health examination process of FSHS consumes public resources and the need for resources will increase significantly in the near future. The services of FSHS will expand to also cover the students of universities of applied sciences (approximately 140,000 students) from the beginning of 2021. This means FSHS will provide student health care services for all higher education students in Finland (approximately 250,000 students). Hence, the number of students to whom the health examination process is provided will approximately double. It is essential to obtain evidence regarding FSHS’s processes to allocate resources effectively.

The main practical implication of this study is the development of the statutory health examination process for higher education students in Finland. Students are especially interested in, and well capable of, using new digital applications. Therefore, the development of the health examination process will focus on digital solutions, for example, the robotization of the eHQ. This study provides information about the functionality of the process, which is needed for further digitalization. Furthermore, we believe the findings will support both health care and the university administration in understanding, more profoundly, the health and welfare requirements of university students.

## Appendix

Multimedia Appendix 1 The continuum of preventive health care services in Finland.

Multimedia Appendix 2 Study ability: the definition of the concept.

Multimedia Appendix 3 The electronic health questionnaire (eHQ) questions.

## Abbreviations

AUDIT: Alcohol Use Disorders Identification Test
eHQ: electronic health questionnaire
FSHS: Finnish Student Health Service
ICD-10: 10th revision of the International Statistical Classification of Diseases and Related Health Problems
ICPC-2: International Classification of Primary Care, Second edition
VIRTA: higher education achievement register
