# Supplementary material for: Screening University Students for Health Checks With an Electronic Health Questionnaire in Finland: Protocol for a Retrospective, Register-Based Cohort Study
Source: JMIR Res Protoc. 2020 Jan 29;9(1):e14535. doi: 10.2196/14535 (PMC7016620; doi:10.2196/14535)
Supplement: Multimedia Appendix 1 [file resprot_v9i1e14535_app1.pdf]

The continuum of preventive health care services in Finland

**Health care service continuum**	**Service providers**	**Population entitled for services**	**Services [a,b]**
Maternity health clinic	Municipalities	All women and couples expecting a child	Preventive health care
Child health clinic	Municipalities	All families with children under school age	Preventive health care
School health care	Municipalities	All primary and secondary school pupils	Preventive health care
Student health care	FSHS for university students, municipalities for others	Upper secondary school students, students in secondary level vocational education, students of universities of applied sciences and basic degree university students	Preventive health care and medical care
Occupational health care	Employer, local authorities or a private provider. Costs covered by employers, partly compensated by The Finnish Social Insurance Institution	All employees (preventive health care)	Statutory preventive health care for all employees, optional medical care services chosen by employers.

[a] Finlex data bank. Government Decree on maternity and child health clinic services, school and student health services and preventive oral health services for children and youth. https://www.finlex.fi/en/laki/kaannokset/2011/en20110338. Accessed 11.3.2019.

[b] Finlex data bank. Government Decree on the principles of good occupational health care practice, the content of occupational health care and the qualifications of professionals and experts. https://www.finlex.fi/en/laki/kaannokset/2013/20130708. Accessed 11.3.2019.
