# Supplementary material for: Screening University Students for Health Checks With an Electronic Health Questionnaire in Finland: Protocol for a Retrospective, Register-Based Cohort Study
Source: JMIR Res Protoc. 2020 Jan 29;9(1):e14535. doi: 10.2196/14535 (PMC7016620; doi:10.2196/14535)
Supplement: Multimedia Appendix 2 [file resprot_v9i1e14535_app2.pdf]

Study ability – the definition of the concept

Study ability is a well-established term in Finland. However, internationally the concept is less known and therefore the definition of study ability is presented in this connection.

Study ability is student's work ability [1]. It is relevant for student well-being, study results, and study progress. Study ability is determined by both individual and study context-related factors. These factors form four interacting components; personal resources, study skills, teaching and study environment [2] (Figure 1). Deficiencies in these components may affect student's study-related well-being. Problems emerging in one area may weaken the individual's potential to cope with problems in another area. On the other hand, personal strengths and resources in one area may compensate for problems in other areas [3]. For a young person, the accumulation of problems is the most devastating [4].

Figure 1. The model for study ability*

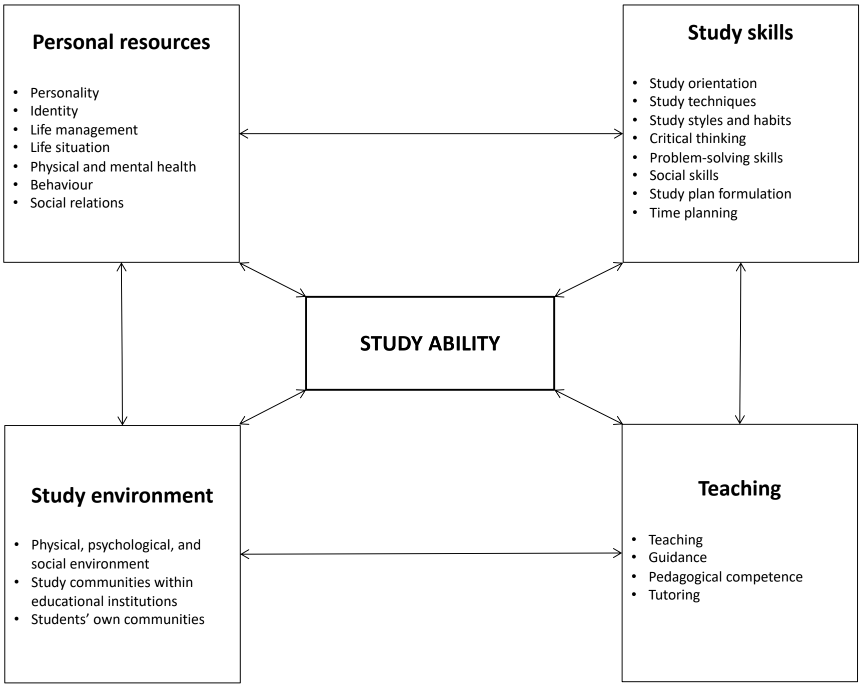

*developed at 2005 by Senior Physician Kristina Kunttu at Finnish Student Health Services (FSHS) and the Finnish Institute of Occupational Health. The model is based on the model for work ability [2].

References

1. Tengland P-A. The Concept of Work Ability. J Occup Rehabil. 2011;21(2):275–85.
2. Sulander J, Romppanen V. Well-being in school and studying: developing a tool for observing student well-being. (Hyvinvointi koulutyössä ja opiskelussa : opiskelijoiden hyvinvointia kartoittavan työkalun kehittäminen). Helsinki: Työterveyslaitos, Työympäristötutkimuksen raporttisarja 26; 2007.
3. Brackney BE, Karabenick SA. Psychopathology and academic performance: The role of motivation and learning strategies. J Couns Psychol. 1995;42(4):456–65.
4. Berg N. Accumulation of Disadvantage from Adolescence to Midlife. A 26-Year Follow-Up Study of 16-Year Old Adolescents. University of Helsinki; 2017.
