# Supplementary material for: Screening University Students for Health Checks With an Electronic Health Questionnaire in Finland: Protocol for a Retrospective, Register-Based Cohort Study
Source: JMIR Res Protoc. 2020 Jan 29;9(1):e14535. doi: 10.2196/14535 (PMC7016620; doi:10.2196/14535)
Supplement: Multimedia Appendix 3 [file resprot_v9i1e14535_app3.pdf]

Researchers comment: This document has been created to describe the questions presented to the students in the electronic health questionnaire (eHQ) program in the academic year 2011-2012. Questions marked with * have a response scale from -10 to + 10.

INSTRUCTIONS

In some of the questions you will be asked to evaluate an issue on a scale from -10 to +10. Positive figures suggest that you experience the matter as favourable and satisfactory, with +10 being the highest possible value. A zero (0) indicates that the matter is neutral for you or that it has not required any special attention. Negative figures suggest that you find the matter problematic or harmful, with -10 standing for the worst possible state.

Please, answer all the questions!

<u>The eHQ questions:</u>

1. Name three most important things in your life at present.

STUDIES

2 *. How enthusiastic are you about your field of studies and studying in general?

3 *. How engaged are you with your studies?

HEALTH STATUS AND HEALTH-RELATED HABITS

4 *. What is the status of your general health?

5. Do you have any chronic or long-term diseases?
|__| No |__| Yes. Please specify:

6. Do you suffer from constant or recurrent medical symptoms?
|__| No |__| Yes. Please specify:

7. When was the last time you had a dental check-up?
|__| 1–2 years ago |__| 3–5 years ago |__| Over 5 years ago

8. Your height _____ cm and weight _____ kg

9 *. How healthy would you say your eating habits are?

10. How many times a day do you eat or drink something (other than water or coffee/tea without sugar)?
|__| 6 times or less |__| 7–10 times |__| More than 10 times

11. Do you exercise?
|__| Yes, for approx. ____ hours per week. Please specify: ___________________
|__| No

12. Do you use alcohol?
|__| No |__| Yes

13. Have you experimented or used any drugs (or taken alcohol and medication at the same time in order to get intoxicated)?
|__| Never |__| Yes, 1–4 times |__| Yes, 5 times or more often

Please specify: ________________________________

14. Do you smoke cigarettes or use any other type of tobacco products (for example, chew tobacco or “snus”)?
|__| No |__| Yes, occasionally |__| Yes, daily

15. How often do you brush your teeth normally?
|__| 2 times a day or more often |__| Once a day |__| Less frequently than once a day

16. At dental check-ups, do you usually have cavities that require fillings?
|__| Never |__| Occasionally |__| Frequently or every time

17. Would you like to discuss any matters related to sexuality, contraception, or sexually transmitted diseases?
|__| No |__| Yes. Please specify: ______________________

SELF-KNOWLEDGE AND PERSONAL RELATIONSHIPS

18 *. What is your state of mind usually?

19 *. How lonely are you?
(Instructions: +10 denotes that you have in your immediate circle people with whom you can spend time and discuss your personal matters and problems; -10 denotes that there are no such people for you.)

20 *. How is the relationship with your parents?

21 *. How do you experience various social situations (for example, giving a presentation)?

22. Do you feel scared about dental care?
|__| Not at all |__| To some extent |__| Very much

23. Is your attitude towards food normal?
|__| Yes |__| No |__| Can not say

24 *. Evaluate your sleep in terms of adequacy and quality (for example, do you fall easily asleep?).

25 *. Evaluate your leisure time in terms of recovery, recreation, and relaxation.

OTHER

26. Do you have any other special issues or problems that you would like to discuss?
|__| No |__| Yes. Please specify: ______________________
